# Water, sanitation and hygiene insecurity predict abscess incidence among people who inject drugs in a binational US–Mexico metropolitan area: A longitudinal cohort study

**DOI:** 10.1016/j.drugpo.2024.104485

**Published:** 2024-06-19

**Authors:** Alhelí Calderón-Villarreal, Daniela Abramovitz, Lourdes Johanna Avelar Portillo, Shira Goldenberg, Shawn Flanigan, Penelope J.E. Quintana, Alicia Harvey-Vera, Carlos F. Vera, Gudelia Rangel, Steffanie A. Strathdee, Georgia L. Kayser

**Affiliations:** a Herbert Wertheim School of Public Health and Human Longevity Science, University of California San Diego (UCSD), San Diego, CA, USA; b School of Public Health, San Diego State University (SDSU), San Diego, CA, USA; c Department of Medicine, Division of Infectious Diseases and Global Public Health, UCSD, San Diego, CA, USA; d Benioff Homelessness and Housing Initiative, School of Medicine, University of California, San Francisco, San Francisco, CA, USA; e Division of Global Health, Herbert Wertheim School of Public Health and Human Longevity Science, UCSD, San Diego, CA, USA; f School of Public Affairs, SDSU, San Diego, CA, USA; g Universidad Xochicalco, Tijuana, Baja California, Mexico; h El Colegio de la Frontera Norte, Tijuana, Baja California, Mexico; i Border Health Commission, Tijuana, Baja California, Mexico

**Keywords:** PWID, WASH insecurity, SSTI, Tijuana, San Diego

## Abstract

**Introduction:**

People who inject drugs (PWID) are at high risk of developing injection-related infections, including abscesses. Access to water, sanitation, and hygiene (WASH) are key human rights and services; yet these services have been underexplored as predictors of abscesses among PWID.

**Methods:**

Longitudinal analysis was employed among a cohort of PWID to determine if WASH insecurity (lack of access) was associated with abscess incidence in the Tijuana, Mexico and San Diego, United States metropolitan area during 24-months of follow-up survey data from 2020 to 2023. We calculated abscess prevalence at baseline and tracked the incidence of new abscesses among individuals without an abscess during the previous visit. Time dependent Cox regression modeling was employed with variance clustered by participant to characterize the relationship between WASH insecurity and abscess incidence.

**Results:**

At baseline, hand hygiene insecurity, bathing insecurity in the previous six months and open defecation in the last week, were reported by 60 %, 54 % and 38 % of participants, respectively; 21 % reported an abscess in the last six months. The incidence of abscesses was 24.4 (95 %CI: 21.1–27.6) per 100 person-years. After adjusting for covariates, the hazard of developing an abscess remained significantly elevated among individuals using non-improved (with risk of contamination) water sources (e.g., surface water) for preparing drugs (adjusted HR [adjHR]: 1.49 [95 %CI: 1.01–2.21], experiencing bathing insecurity (adjHR: 1.59 [95 %CI: 1.12–2.24]) and open defecation (adjHR: 1.65 [95 %CI: 1.16–2.35]).

**Conclusions:**

PWID in the Tijuana-San Diego metropolitan area reported facing high rates of insecurity accessing WASH services. Abscess incidence was higher (four to nine times) than observed rate among PWID cohorts in other settings. Access to continuously available toilet facilities, bathing infrastructure, and safe water sources for preparing drugs for injection could prevent abscesses among PWID. Accessible WASH infrastructure should be ensured among PWID communities and promoted as a key component of harm reduction infrastructure.

## Introduction

People who inject drugs are at high risk of developing infection-related diseases associated with increased morbidity and mortality (Kolla et al., 2020). Wounds and skin and soft tissue infections (SSTI) are common complications of injection drug use among people engaged in drug injection and are the most common cause of emergency department visits and medical care among this population (Calderón-Villarreal et al., 2022; Chambers, 2021; Colledge et al., 2020; Doran et al., 2020; Pollini et al., 2010; Sanchez et al., 2021). Abscesses, defined as a “collection of pus that has built up within the tissue of the body” or a “tender, fluctuant (compressible), palpable lesion with erythema” (i.e., fluctuant red nodule) (Lipsker, 2021), are the most common types of SSTI (Ozga et al., 2022; Pieper, 2019; Wright et al., 2020).

If not treated, SSTI can lead to life-threatening complications such as necrotizing fasciitis, gangrene, amyloid A amyloidosis, deep vein thrombosis, endocarditis, septic arthritis, septicemia and death (Colledge et al., 2020; Doran et al., 2020; Harris et al., 2020; Harris et al., 2018; Larney et al., 2017; Noroozi et al., 2019; Ozga et al., 2022; Wright et al., 2020). Abscesses can cause a significant level of pain, discomfort, scarring and disabilities, which negatively impact the capacity to work and also contribute to stigma (Calderón-Villarreal et al., 2022; Harris et al., 2020; Noroozi et al., 2019; Ozga et al., 2022; Wright et al., 2020). Further, wounds and abscesses can also contribute to increased use of substances, as individuals may seek to reduce SSTI-related pain (Sanchez et al., 2021).

People who inject drugs need water not only for drinking and handwashing, but also for preparing injection drugs, rinsing syringes and cleaning wounds and abscesses (Calderón-Villarreal et al., 2024b; Harris et al., 2020; Sanchez et al., 2021). Water sources can be ‘improved’ if they have the potential to deliver safe water by design (e.g., piped and bottled water) (WHO, 2020). Qualitative studies suggest that individuals engaged in drug injection tend to use the safest water available to them for preparing their drugs (Calderón-Villarreal et al., 2022; Gilbert et al., 2019). However, when improved water is not available or a person is experiencing withdrawal, individuals may use ‘non-improved’ (with high risk of contamination) water sources, such as surface water (e.g., river, canal, ditch), soda, alcoholic beverages, fruit juice, toilet water, saliva and puddle water (Calderón-Villarreal et al., 2024a; Baltes et al., 2020; Calderón-Villarreal et al., 2022; Harris et al., 2020; Otiashvili et al., 2016).

Lack of access to improved water, sanitation and hygiene services (i.e., WASH insecurity) and poor access to improved water for cleaning wounds is common among individuals who inject drugs— especially for those who are unhoused (Calderón-Villarreal et al., 2024a; Calderón--Villarreal et al., 2022; DeMyers et al., 2017; Leibler et al., 2019,Flanigan & Welsh, 2020; Sanchez et al., 2021) and who engage in sex work (Calderón-Villarreal et al., 2024b; Phillips-Howard et al., 2024; Wurcel et al., 2018). People who inject drugs in the Tijuana-San Diego region have lower WASH access than the national averages in Mexico and the US, and that is lower than international standards (Calderón-Villarreal et al., 2024b). Homelessness has been significantly associated with WASH insecurity among people who engage in substance use in the US–Mexico metropolitan area (Calderón-Villarreal et al., 2024b). Similarly, among people who inject drugs and who menstruate in the same cohort population, housing status and sex work status were associated with insecurity accessing hygiene services (water for handwashing, and handwashing facilities with soap and water) (Calderón-Villarreal et al., 2024a). Further, a study in Kenya reported sub-optimal WASH services in most female sex work venues (e.g., 34 % had no adequate WASH facilities) (Phillips-Howard et al., 2024).

Insecurity accessing improved water sources and hand hygiene (water and soap for handwashing) are thought to be a common cause of skin irritation and can introduce infection when preparing the site of injection (Robertson et al., 2021; Sanchez et al., 2021). These experiences of WASH insecurity decrease quality of life, wellbeing and dignity, and exacerbate social exclusion and health risks among vulnerable groups, including people engaged in substance use (Avelar Portillo et al., 2023; Avelar Portillo et al., 2024; Calderón-Villarreal et al., 2022; Neves-Silva et al., 2018; Uddin et al., 2016).

Securing WASH access is a core concern of public health, epidemiology and rural and urban planning, and is considered “the most crucial major urban environmental infrastructure necessary for human survival” (Calderón-Villarreal et al., 2022; Rosenberg, 1966). Access to WASH services is a human right that is interdependent and inseparable from other human rights; the violation of one affects all others, generating inequities and harm to health and wellbeing (Avelar Portillo et al., 2023; Avelar Portillo et al., 2024; Neves-Silva et al., 2018). The right to WASH is part of the right to quality of life and is directly linked to the right to food, health, and housing (Neves-Silva et al., 2018; UN General Assembly 2010). Likewise, WASH insecurity among people engaged in drug injection has been linked to environmental structural violence and increases the risk of infectious diseases, human rights violations, stigma, exclusion and discrimination, jeopardizing basic life needs (Calderón--Villarreal et al., 2022; Neves-Silva et al., 2018; WHO 2015). The development of abscesses represents a potent example of these deleterious outcomes.

Existing studies have estimated that between 7 % and 65 % of people who inject drugs have experienced SSTI in the past 12 months, with 40–67 % ever having reported an occurrence of prior SSTI (half of which led to hospitalization) (Doran et al., 2020; Dwyer et al., 2009; Harris et al., 2020; Harris et al., 2018; Noroozi et al., 2019; Ozga et al., 2022; Wright et al., 2020). SSTI incidence was estimated as 2.8 per 100 person-years (py) among a sample of 2444 people engaged in drug injection from the Swedish Prison and Probation Service in 2018 and 6.1 per 100 py among a sample of 1083 individuals engaged in drug injection randomly selected from an supervised injection facility in Vancouver, Canada (Dahlman et al., 2018; Lloyd-Smith et al., 2010). Abscess prevalence has been associated with a variety of factors, such as female sex, age, injection technique (e.g., intravenous vs intramuscular), injection site (e.g., neck, groin), sex work—particularly among women—and injection frequency (Baltes et al., 2020; Calderón-Villarreal et al., 2022; Chambers, 2021; Colledge et al., 2020; Lewer et al., 2020; Doran et al., 2020; Pieper, 2019; Pollini et al., 2010; Wright et al., 2020).

The United States (US)-Mexico border region of Tijuana and San Diego has a large population of people engaged in drug injection, many of whom experience additional vulnerabilities such as homelessness (West et al., 2020,40). An estimated 6400 to 10,000 people who inject drugs live in Tijuana (Fraser et al., 2021) and 21,800 to 35,000 live in San Diego (Lewis & Asmus, 2019). Previous studies have found that lifetime abscess prevalence is high among people who inject drugs in Tijuana (46 % in 2010) and San Diego (73 % in 2015) (Armenta et al., 2015; Pollini et al., 2010). In a previous study among the same study cohort, WASH insecurity was common and associated with housing status and city of residence (Calderón-Villarreal et al., 2024b). Access to basic WASH services in this cohort was lower than international standards and national averages for both countries (Calderón-Villarreal et al., 2024b). Also, among a study of Tijuana’s unhoused individuals who inject drugs who reside inside the Tijuana River canal, abscesses (*cuerazos*) or skin infections were the most common WASH-related condition reported in the previous week (47 %), and were significantly associated with use or contact with contaminated surface water (Calderón-Villarreal et al., 2022). Similarly, a study in San Diego found that among unhoused individuals living along the San Diego River (many of whom inject drugs), numerous health concerns were observed, including open sores and infections located at injection sites (Flanigan & Welsh, 2020).

Despite the high burden of disease from SSTI among people who inject drugs and the likely link to WASH practices, limited studies have focused on this critical set of risk factors, especially using longitudinal data (Summers et al., 2017; Wright et al., 2020). Few existing studies have examined cross-sectionally the intersection between substance use and environmental health among individuals who inject drugs, and suggest there is an increased risk of wounds, abscesses, Hepatitis C complications, multidrug resistant bacterial infections, septic shock and myocarditis associated with WASH insecurity (Calderón-Villarreal et al., 2022; Leibler et al., 2019; Sanchez et al., 2021; Verbyla et al., 2021).

A better understanding of the relationship between WASH insecurity as a key driver of injection-related abscess incidence among people who inject drugs is critical for reducing harms associated with infectious disease and improving living conditions for these communities (Ballard et al., 2022; Calderón-Villarreal et al., 2022). Herein, we assessed whether WASH insecurity factors were associated with higher incidence of injection-related abscesses over a 24-month period among a binational cohort of people who inject drugs in the Tijuana and San Diego metropolitan area.

## Methods

This study leveraged data from the *La Frontera* study and *LinkUp* sub-study to examine the association between WASH insecurity and incidence of injection-related abscesses over a 24-month period. *La Frontera* is a prospective cohort study designed to study incidence and predictors of HIV, HCV, and overdose among people who inject drugs who live in the binational metropolitan area of Tijuana, Baja California, Mexico and San Diego, California (CA), US (Strathdee et al., 2021). *LinkUp* is a *La Frontera*’s sub-study, which aimed to evaluate the impact of a peer-led behavioral intervention to improve COVID testing and vaccination among people who inject drugs in San Diego only. This analysis included data from the baseline, six, 12-, 18- and 24-month follow-up survey visits conducted between October 2020 to August 2023.

### Study population

*La Frontera* recruitment (cohort 1) began in 2020 and was re-opened in 2022 to accommodate a sub-study, *LinkUP* (cohort 2). The *La Frontera* cohort recruitment was open from October 2020 to September 2021, and recruitment for the *LinkUP* was open from March to June 2022. For both cohorts, data was collected by trained interviewers in the participants’ language of preference (English or Spanish) using street outreach and mobile vans, as previously described (Bazzi et al., 2022; Strathdee et al., 2021). Cohort 1 included participants residing in Tijuana and San Diego whereas only participants residing in San Diego were included in the sub-study cohort 2. To reduce the risk of acquiring SARS-CoV-2, interviews were done outside in tents with plexiglass partitions and full personal protective equipment. All protocols were reviewed and approved by UCSD Risk Management. More details on recruitment protocols can be found in Strathdee et al. (2021). Recruitment interruptions due to COVID-19 outbreaks took place only for a two-week period.

In both cohorts, eligibility criteria were age ≥ 18, report of injecting drugs within the last month—verified in limited instances by inspecting injection marks, and living in Tijuana or San Diego (Strathdee et al., 2021). A seven-minute screener survey programmed into a tablet enabled screening via computer-assisted programmable interviews (CAPI) to exclude ineligible participants. Of the *N* = 720 participants (cohort 1 = 612 and cohort 2 = 108) found to be eligible for *La Frontera, N* = 647 (89.9 %) participants completed at least two visits and were included in this study analysis (cohort 1 = 553, cohort 2 = 94). We had up to 3-month window periods where we allowed participants to be late for a visit, after which time they were recorded as having missed that visit. Longitudinal data included a baseline survey and five (2020–2023 – cohort 1) or four (2022–2023 – cohort 2) follow-up visits. Each survey took approximately 45 min and was enumerator administered. The baseline sample included *n* = 647 participants for visit #1, visit #2 (6-month) included *n* = 601, visit #3 (12-month) included *n* = 573, visit #4 (18-month) included *n* = 488 and visit #5 (24-month) included *n* = 440 participants, 69.6 % from San Diego, US and 30.4 % from Tijuana, Mexico. Characteristics of excluded and included participants are described in Supplemental Table 1.

### Health outcomes

In this analysis, the outcome was obtained via self-report of having at least one abscess (*cuerazo* in Spanish slang) at a location on their bodies where they inject drugs in the last six months. Abscesses were categorized dichotomously at the five follow-up time points: baseline, 6-month, 12-month, 18-month and 24-month visits.

### WASH insecurity variables

Seven time-varying binary WASH insecurity variables were included as abscess incidence predictors: 1) use of non-improved water as the main water source for injecting drugs, 2) use of non-improved water as the main water source for cleaning wounds, 3) use of non-improved water as the main water source for handwashing, 4) basic hand hygiene insecurity 5) bathing insecurity, 6) basic sanitation insecurity and 7) open defecation practice. Variables were operationalized based on the WHO and UNICEF’s Joint Monitoring Program (JMP) definitions (WHO, 2020).

The use of ‘non-improved’ water sources was defined according to the main (primary) water source for injecting drugs, cleaning wounds and handwashing reported by participants in the past six months. Non-improved water sources included bottled water taken from the trash, water treatment plant discharge into riverbeds, pet water bowls, water directly from a river, pond, stream or irrigation canal, *lloraderos* (i.e., unprotected water spring tubes next to the Tijuana River canal) (Calderón-Villarreal et al., 2022), and the use of ‘no water’ sources such as soda, alcohol beverages, wet wipes, hand sanitizer, ointments, or aloe vera instead of water (Calderón-Villarreal et al., 2024b; JMP, UNICEF, WHO 2023).

Basic hand hygiene insecurity was defined as not having a handwashing facility with soap and water always available in the past six months (WHO, 2020). Bathing insecurity was defined as having less than five baths/showers per week and/or bathing using non-improved water sources in the past six months. The number of showers was described as a continuous variable.

Basic sanitation insecurity was defined as the use of ‘non-improved’ toilet facilities (e.g., bucket latrines, or not having access to toilet facilities/open defecation) as their main form of sanitation in the past six months (WHO, 2020). Open defecation was described as the disposal of human feces in fields, forests, bushes, open bodies of water, beaches, and other open spaces or with solid waste in the last week. Open defecation was described as dichotomous (at least once in the last week) and for those who reported open defecation, the number of times defecating in the open was described as a continuous variable.

### Covariates

Demographic covariates of interest included city of residence (Tijuana/San Diego), gender identity, and housing status. Gender identity was classified as man, woman, trans men, trans women, and nonbinary. For statistical analysis, gender was classified as men (*cis*, trans men and nonbinary individuals who reported man as their sex at birth) and women (*cis*, trans women and nonbinary individuals who reported woman as their sex at birth). Housing status was a time varying variable based on the main places used for sleeping in the past six months. Permanent housing was defined as those sleeping at their parent’s home, own apartment, with their spouse’s/sex partner, or friends, whereas homelessness included sleeping in sheltered and unsheltered locations (Orange County 2022; US Department of Housing & Urban Development 2021). Sheltered homelessness was defined as those who reported living in shelters, temporary rented rooms, and other institutions (e.g., correctional institution, drug treatment center and medical care facility). Unsheltered homelessness included sleeping in a car, bus, truck, or other vehicle, abandoned building, on the streets, beach, parks, canal, woods and shooting gallery.

Other fixed covariates included gender, years of education and cohort, and time varying variables age, engagement in sex work, days since last injection and most common site of the body used for injecting. Age and years of education were described as continuous and dichotomous variables (<45 vs 45+ years of age, <10 vs 10+ years of education, respectively). Sex work was defined as a source of income and described as ‘prostitution or sex work’ in the past six months. Number of days since last injecting drugs was described as a continuous and dichotomous variable (same day vs 1+ days). Most common body injection site included: 1) safest sites – arms, legs, hands, feet and between fingers and 2) most dangerous sites – neck, inguinal area, armpits and temple (Karimi et al., 2014; Robertson et al., 2021).

### Statistical analysis

Descriptive statistics were calculated for potential confounders and WASH insecurity variables at baseline. For categorical variables we reported frequencies and proportions. For continuous variables we reported mean and standard deviation (SD) or medians and IQR according to their distributions.

Longitudinal imputation methods were implemented to manage missing values. WASH insecurity indicators with missing values were imputed using the last value carried forward/backwards method, utilizing the previous or following value by participant (mostly to fill missing from skip logic errors) (Twisk, 2013). The frequency of missing values by WASH insecurity variable is shown before and after imputation Supplemental Table 2. This process was limited to 12 months, so values were not extended past 12 months from their collection date. Imputation for missing values of abscess status were calculated using the mode of all 0 (absent) and 1 (present) value across all available time-points for a given individual. In rare instances when the average value for an individual was exactly 0.5 (in a 0–1 range), the imputed value was assigned the population average.

Abscesses were described by frequency and incidence rate. Abscess frequency (prevalence) was described at baseline and as a recurrent event. To calculate incidence rate (reflecting only new-onset symptoms) *only* participants who did not have an abscess in the previous visit were included for each period of incidence calculation. Given that abscesses may persist for long periods of time, this method helped ensure that the measured incidence rate values only reflect de-novo infections and that we did not misclassify past abscesses that persisted as being incident cases. To compute incidence rate, we defined person-time at risk, which served as the denominator, as all six-month windows of time during which the individual did not have an abscess during the previous visit. All participants’ time at risk were summarized to obtain cumulative person-time at risk. The abscess incidence rate was estimated as the number of participants experiencing new-onset abscesses in the prior six-month period divided by the cumulative person-time at risk.

We used time-varying Cox regression models to estimate the effect of WASH insecurity variables on abscess incidence. We first conducted bivariate Cox regressions, reporting hazard ratios (HR) and used the results to inform the multivariable model. We included demographic variables previously shown to be associated with abscesses and/or WASH insecurity from existing literature (Avelar Portillo et al., 2024; Baltes et al., 2020; Calderón-Villarreal et al., 2022; Lewer et al., 2020; Doran et al., 2020; Pieper, 2019; Pollini et al., 2010; Wright et al., 2020; Wurcel et al., 2018). Only WASH insecurity and abscess-related covariables with significant values (*p* < 0.05) in bivariate time-varying Cox analyses were chosen to be included in the final model. No other method to remove predictors was employed. Five of the seven WASH variables, days since last injection and injects in more dangerous body areas had the strongest association with abscess incidence and were included in the adjusted hazard regression model. The association between the aforementioned variables and the abscess incidence rate was reported as adjusted hazard ratios (adjHR) (Therneau et al., 2023). All Cox models included robust variance clustered—by ID—on study subject (i.e., multiple outcome events were potentially included per person) (Therneau et al., 2023). We assessed collinearity among WASH variables and homelessness (Supplementary Fig. 1) as well as the assumption of proportionality in the final Cox model (Supplemental Table 3). Associations were reported as HRs with 95 % confidence interval (95 % CI). All data analyses were performed using R (Version 4.3.1).

### Ethics statement

The study protocols received ethics approval from the institutional review boards at the University of California, San Diego (UCSD) (IRB# 191390) and Xochicalco University in Mexico. Only participants that consented were enrolled in the study. Study activities were carried out in accordance with IRB guidelines. Monetary reimbursements ($20 USD) were provided to enrolled participants at each visit.

## Results

A total of 2749 participant visits and 784 py at risk were included in this analysis (median: three visits per person; inter quartile range [IQR]: 2–4). Of 647 participants who completed at least two visits, at baseline 448 resided in San Diego and 196 in Tijuana. The median age was 43 years (IQR=35–52 years), and participants had a median of 11 years of education (IQR=8–12). Seventy-one percent were *cis* men, 28 % *cis* women and 1 % identified as transgender or nonbinary. Most had experienced homelessness at least once in the last six months (72 %). At baseline 33 % reported living in permanent housing, 21 % experienced sheltered homelessness, and 46 % experienced unsheltered homelessness. Eight percent of sample reported having a source of income related to sex work in the past six months. Participant characteristics are included in Table 1.

At baseline, 64 % of participants had injected drugs on the day of the survey. Most common body sites used for injecting in the last six months were ‘safer sites’ (82 %), such as arms (65 %) and legs (11 %), but 15 % used ‘more dangerous’ sites, such as the neck (13 %).

### WASH insecurity

In terms of WASH insecurity at baseline, the use of non-improved water in the past six months was reported by 8 % for injecting drugs, 6 % for cleaning wounds and 5 % for handwashing. Basic hand hygiene insecurity was reported by 59 %. Bathing insecurity (i.e., <5 showers in the last week and/or using non-improved water sources) was reported by 54 %, with an average of four (±3) showers in the last week. Basic sanitation insecurity, defined as the use of non-improved main toilet facilities in the past six months, was reported by 17 % of participants. Open defecation during the previous week was practiced by 38 %. Of those practicing open defecation, median frequency was four times/week (IQR=2–7), ranging from one to 24 times. Frequency of WASH insecurity at baseline and follow-up visits are reported in Table 2.

### Abscess frequency, incidence and predictors of incident abscesses

At baseline, 21 % of participants reported having an abscess in the last six months. Over the 24-month follow-up period, the abscess incidence rate was 24.4 (95 %CI: 21.1–27.6) per 100 py and the cumulative incidence was 31.7 per 100 participants, representing 191 new abscesses. Among participants residing in Tijuana the incidence rate was 23.6 (95 %CI: 19.7–27.4) new abscesses and in those residing in San Diego 26.1 (95 %CI: 20.1–32.2) new abscesses per 100 py.

**Fig. 1.**
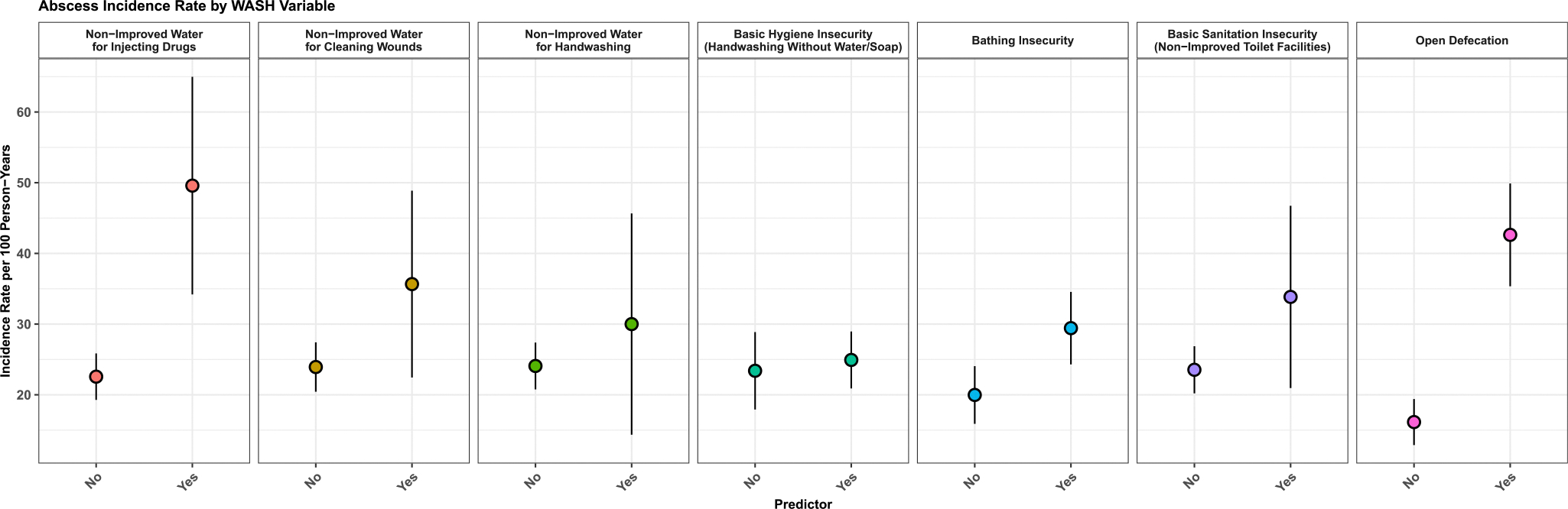
Crude abscess incidence rate per 100 person-years by WASH insecurity variables among people who inject drugs in the Tijuana-San Diego metropolitan area, *La Frontera* cohort study, 2020–2023 (*N* = 647). Fig. 1 summarizes abscess incidence rates per 100 person-years stratified by key WASH variables. The incidence rates are crude, not adjusted for the effect of other variables. All points are shown with 95 % confidence intervals. The significance of each WASH variable can be assessed using the unadjusted hazard ratios shown in Fig. 2.

The abscess incidence rate by WASH variables is described in Fig. 1 and covariates are described in Supplemental Table 4. Abscess incidence was higher among those who used non-improved water sources for preparing drugs for injection (incidence rate: 49.6 [95 %CI: 34.2–65.0] vs those who used improved water sources: 22.6 [95 %CI: 19.3–25.8]), those who experienced bathing insecurity (incidence rate: 29.4 [95 %CI: 24.3–34.6] vs those who had bathing access: 20.0 [95 %CI: 15.9–24.1]) and those who practiced open defecation (incidence rate: 42.6 [95 %CI: 35.4–49.9] vs those who did not practice open defecation: 16.1 [95 %CI: 12.9–19.4]). The abscess incidence rates did not significantly differ by age, years of education, city of residence, gender identity, housing status and reported sex work.

**Fig. 2.**
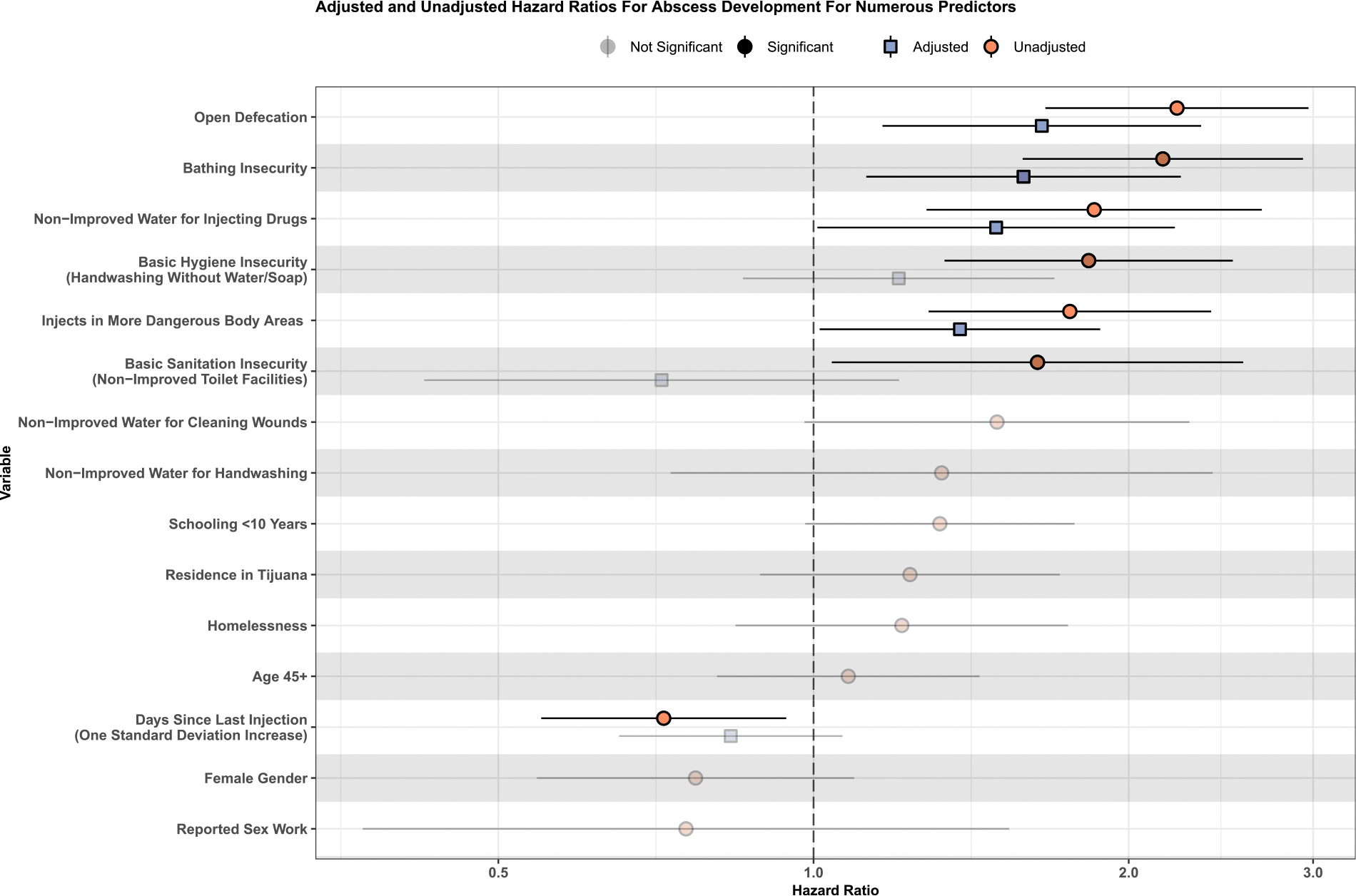
Hazard Ratios from Cox Models for Abscess Development in Past Six Months for All Considered Predictors among people who inject drugs in the Tijuana-San Diego metropolitan area, *La Frontera* cohort study, 2020–2023 (*N* = 647). Figure 2 summarizes all Cox models used to assess predictors of abscess formation. Unadjusted models were employed first, assessing the association of each variable with abscess formation. A single adjusted model was subsequently used, including all variables found to be statistically significant in the first round of models. Adjusted vs. unadjusted HRs are shown with point shape and fill color. Statistically significant vs not statistically significant at the 0.05 level was shown with the intensity of the point color. 95 % CI are shown for all HRs, and a vertical line shows 1.0, the point of non-significance.

Unadjusted and adjusted HR and 95 %CI are described in Fig. 2 and Supplemental Table 4. The unadjusted hazard of developing new abscesses was significantly associated with the use of non-improved water sources for preparing drugs for injection (HR: 1.85 [95 %CI: 1.28–2.68]), basic hand hygiene insecurity (HR: 1.83 [95 %CI: 1.33–2.51]), bathing insecurity (HR: 2.16 [95 %CI: 1.58–2.93]), basic sanitation insecurity (HR: 1.64 [95 %CI: 1.04–2.57]), open defecation (HR: 2.22 [95 %CI: 1.67–2.97]), days since last time of injection (HR: 0.72 [95 %CI: 0.55–0.94]), and injecting in more dangerous body areas (HR: 1.76 [95 %CI: 1.29–2.40]).

After adjusting for potential confounders, the hazard of developing a new abscess remained significantly associated with insecurity accessing improved water sources for preparing drugs for injection (adjHR: 1.49 [95 %CI: 1.01–2.21]), bathing insecurity (adjHR: 1.59 [95 %CI: 1.12–2.24]) and open defecation (adjHR: 1.65 [95 %CI: 1.16–2.35]). The cohort variable (cohort 1 vs cohort 2) had no significant association in bivariate analyses and controlling for cohort in multivariable analyses did not change the direction or significance of other associations. We ran a series of sensitivity analyses using 1) a complete case approach and 2) imputing only WASH variables. In each case, the magnitude and direction of all coefficients for the WASH insecurity variables remained unchanged. In both sensitivity analyses the overall estimated abscess incidence remained almost unchanged at 23.7 versus 24.4 per 100 person-years.

## Discussion

In this study, as previously reported (Calderón-Villarreal et al., 2024b), we found that WASH insecurity was frequent, abscess prevalence and incidence were high, and open defecation, bathing insecurity and the use of non-improved water sources for preparing drugs were predictors of abscess incidence among a binational cohort of people who inject drugs in the Tijuana-San Diego metropolitan area. The risk of developing injection-site abscesses was high regardless of housing status and city of residence. Homelessness had positive, albeit weak correlations with the WASH insecurity variables included in our study, but it cannot fully explain WASH insecurity or abscess incidence. Other studies of WASH and SSTI have been cross-sectional in nature and focused on more limited sets of WASH indicators. We provide the most comprehensive, and first longitudinal evidence, that WASH access represents a key set of determinants of abscess incidence among people who inject drugs.

Prevalence and incidence of abscesses among individuals who inject drugs appears to vary depending on the context and period studied, and is likely influenced by a myriad of environmental and individual factors (Fink et al., 2013; Larney et al., 2017). At baseline, we found that one in five participants reported having an abscess in the prior six months, which was similar to that observed in a study among people who inject drugs in Canada (21.5 %) in 2005 (Lloyd-Smith et al., 2005). However, the incidence of abscesses in our study (24.4/100 py) was about four to nine times higher than rates observed in other cohorts of individuals engaged in drug injection in Canada in 2010 (6.1/100 py) (Lloyd-Smith et al., 2010) and Sweden in 2017 (2.8/100 py) (Dahlman et al., 2018). In our study, participants were recruited using targeted sampling not linked with public or private services (Strathdee et al., 2021), while other cohort studies enrolled participants based on their participation in institutions (public prison) or formal programs (supervised injection facilities) (Dahlman et al., 2018; Lloyd-Smith et al., 2010). Drug policies, criminalization of substance use and availability of harm reduction programs and supervised injection facilities in study locations could influence differences in incidence rates. Additionally, our study was conducted during the COVID-19 pandemic, and in the years since those studies were published, a housing crisis in California may be increasing rates of homelessness broadly among individuals engaged in substance use, which may be worsening WASH access and abscess incidence.

Limited studies have described—or assessed associations with—the use of non-improved water sources for preparing drugs, such as surface water or saliva (Calderón-Villarreal et al., 2024b; Baltes et al., 2020; Calderón-Villarreal et al., 2022; Harris et al., 2020; Otiashvili et al., 2016). We found that individuals using non-improved water sources for preparing drugs for injection (one in every 12 participants) had the highest incidence of abscesses and this practice was a robust predictor of abscess incidence. Water sources used for preparing drugs for injection has been associated with abscess prevalence (Baltes et al., 2020), but in our knowledge, no previous study has explored its relationship with abscess incidence. Although the use of non-improved water sources is not preferred or common among people who inject drugs, multiple factors such as environmental structural violence and insecurity accessing sterile/improved water sources for injection can render it an unfortunate necessity (Calderón-Villarreal et al., 2022; Harris et al., 2020). People use water in the preparation of a wide range of drugs for injection (e.g., heroin, fentanyl, methamphetamine) (Harris et al., 2020). For instance, individuals who injected heroin and crack cocaine together usually add water (or an alternative to water) in two distinct moments during the drug preparation: 1) water to mix heroin, which is heated and 2) cold water used before mixing with crack cocaine. Therefore, in the context of rapidly rising polysubstance use (especially of fentanyl, xylazine and stimulants) (Friedman & Shover, 2023; Friedman et al., 2022), more research on the specific water types and quality used during drug preparation is needed.

There is a large research gap related to bathing insecurity (e.g., frequency and facilities access/functionality/safety) among people who inject drugs, particularly important among those experiencing housing instability. We found that lack of access to body hygiene (bathing insecurity) was an independent predictor of abscess incidence. Similarly, open defecation and sanitation insecurity have been unexplored as predictors of abscesses. We found open defecation was independently associated with abscess incidence. Broader infectious disease and substance use-related research and policies focused on open defecation and continuously available toilet facilities is needed among communities of people engaged in substance use—and those who experience housing instability.

In our study, water used for cleaning wounds, handwashing and basic hand hygiene insecurity were not found to be significant predictors for abscess incidence. However, literature has reported contradictory results regarding of water sources use or contact and skin cleaning prior injection associations with SSTI/abscesses prevalence. In a study conducted by some of the authors in our study (ACV, SAS and GLK) among 84 unhoused individuals who inject drugs and lived in the Tijuana River canal in Tijuana, Mexico, we found that people who used or were in contact with surface water were two times more likely to have SSTI than those who did not (Calderón-Villarreal et al., 2022). Similarly, in San Francisco, Dahlman et al.’s (2015) study among 201 people who inject drugs reported an association between infrequent skin cleaning before injection and SSTI prevalence (Dahlman et al., 2017). While these studies identified an association between forms of WASH insecurity and prevalence of SSTI, Larney et al. (2017) conducted a systematic review that included 29 cross-sectional studies of SSTI prevalence among individuals engaged in drug injection in different contexts, reporting no significant association between SSTI and skin cleaning prior to injection or handwashing prior to injection (Larney et al., 2017). This remains an important area for further study to parse out the contradictory results.

There are limitations to this study, including attrition due to challenges in following participants that are experiencing homelessness or incarceration, which could attenuate some associations. It is possible that some WASH outcomes, such as open defecation, were influenced by COVID-19 related measures, for example, if public restrooms were less available than outside of the pandemic period. On the other hand, our decision to limit incidence calculations to individuals who did not have an abscess in the prior 6-month period—although necessary to avoid overcounting—may have led to underestimating the true incidence rate. There is also a possibility of misclassification bias since participants self-reported having abscesses and some could represent other types of SSTI, such as a wound without a collection of pus (e.g., cellulitis) (Lipsker, 2021). Further, we classified water sources according to the JMP definitions for drinking water; however, this classification is not tailored for people who inject drugs or who experience homelessness. We also used and defined water insecurity based on the JMP definitions instead of other scales to measure water insecurity (Strathdee et al., 2012), as these have been tested globally. However, we did expand them to dimensions such as shower frequency and water for drug preparation and injection—which we argue are critical for the study population and to similar populations across the globe. We did not test the quality of the water people reported using, thus we cannot determine if the water people used was safe or continuity (continuously available 24-h per day).

More than half of individuals experiencing SSTI will have at least one recurrent infection in their lifetime impacting their quality of life and limiting their mobility (McNeil & Fritz, 2019). SSTI-related healthcare has financial implications for both the patient and health care systems (~190 million USD per year) (Chambers, 2021; Cross, 2013). Further, about half of people who inject drugs experience more than one injection related injury and disease at a time (Colledge et al., 2020), increasing complications and costs.

WASH infrastructure that provides access to improved water sources, showers, handwashing, and 24-h sanitation facilities is needed to prevent the incidence of abscesses and related complications for people who inject drugs. Local governments and civil society play an important role as actors that can potentially modify the structural barriers that limit people who use drugs’ access to continuously available WASH services. We argue that WASH should be considered a key component of harm reduction infrastructure, research, and policy. For instance, water provided by harm reduction services often is the only improved water source for drinking, preparing drugs and cleaning wounds among communities of people who inject drugs. Harm reduction strategies and other local/state level programs integrating WASH services, may decrease abscesses (Chambers, 2021). Also, public WASH facilities should be continuously open and functional for everyone. Further, among those who inject drugs and who experience housing instability, transitional housing (such as shelters), ‘Housing First’ programs (affordable housing not conditioned on behavioral change) (O’Sullivan et al., 2021) and mobile hygiene services could reduce WASH insecurity. Mobile hygiene services can hold up the principle of ‘Radical Hospitality’—the idea to “*treat those who often feel invisible and dehumanized with an extraordinary level of respect and care to restore dignity and unlock the opportunities that come with being clean*” (LavaMae 2022; Zelayandia & Leon, 2020). This is highly compatible with the existing ethos of many harm reduction programs: receiving people as they are and helping them improve their quality of life as much as possible.

## Conclusions

In this cohort study of people who inject drugs in the Tijuana-San Diego metropolitan area, we document high incidence of insecurity accessing WASH services. Abscess incidence was about four to nine times higher than rates observed among individuals who inject drugs in other contexts and was independently associated with the WASH insecurity variables of open defecation, body hygiene insecurity, and the use of non-improved water sources for preparing drugs. Our findings demonstrate that WASH insecurity variables are important and understudied predictors of abscess incidence that should be incorporated into further infectious disease studies among people who inject drugs. Access to basic WASH infrastructure should be ensured and championed as a key component of harm reduction infrastructure to prevent the incidence of abscesses and infectious diseases, to decrease stigma, and address environmental injustices among communities of people who inject drugs.

## Supplementary Material

Supplementary material

Supplementary material associated with this article can be found, in the online version, at doi:10.1016/j.drugpo.2024.104485.

## Figures and Tables

**Table 1:** Baseline characteristics of people who inject drugs by baseline abscess status, *La Frontera* cohort study, Tijuana-San Diego metropolitan area 2020–2023 (*N* = 644).

Variable	With Abscess (*N* = 134)	Without Abscess (*N* = 510)	Overall (*N* = 644)

**Age**			
Mean (SD)	43.1 (10.8)	43.5 (10.7)	43.4 (10.7)
Median [IQR]	41.0 [35.0, 51.0]	43.0 [35.0, 52.0]	43.0 [35.0, 52.0]
45+ years	50 (37.3 %)	237 (46.5 %)	287 (44.6 %)
<45 years	84 (62.7 %)	273 (53.5 %)	357 (55.4 %)
**Years of Education**			
Mean (SD)	10.0 (3.4)	10.0 (3.1)	10.0 (3.2)
Median [IQR]	11.0 [8.0, 12.0]	11.0 [8.0, 12.0]	11.0 [8.0, 12.0]
<10 years	48 (36.1 %)	204 (40.5 %)	252 (39.6 %)
10+ years	85 (63.9 %)	300 (59.5 %)	385 (60.4 %)
**City of Residence**			
Tijuana	44 (32.8 %)	152 (29.8 %)	196 (30.4 %)
San Diego	90 (67.2 %)	358 (70.2 %)	448 (69.6 %)
**Gender**			
Cis men	106 (79.1 %)	354 (69.4 %)	460 (71.4 %)
Cis women	28 (20.9 %)	150 (29.4 %)	178 (27.6 %)
Trans gender or nonbinary	0 (0 %)	6 (0.9 %)	6 (0.9 %)
**Housing Status**			
Permanent housing	36 (26.9 %)	178 (34.9 %)	214 (33.2 %)
Homelessness			
Sheltered	15 (11.2 %)	121 (23.7 %)	136 (21.1 %)
Unsheltered	83 (61.9 %)	211 (41.4 %)	294 (45.7 %)
**Sex Work Status**			
No sex work	122 (91.0 %)	469 (92.0 %)	591 (91.8 %)
Sex work	12 (9.0 %)	41 (8.0 %)	53 (8.2 %)
**Days Since Last Injection**			
Mean (SD)	1.2 (3.8)	1.7 (4.2)	1.6 (4.1)
Median [IQR]	0 [0, 1.0]	0 [0, 1.0]	0 [0, 1.0]
**Most Common Site of Injection**			
Safer			
Sites Arms	93 (72.7 %)	326 (65.9%)	419 (67.3%)
Feet	2 (1.6 %)	10 (2.0 %)	12 (1.9 %)
hands	6 (4.7 %)	19 (3.8 %)	25 (4.0 %)
Legs	14 (10.9 %)	54 (10.9 %)	68 (10.9 %)
Between Fingers	0 (0 %)	2 (0.4 %)	2 (0.3 %)
More Dangerous Sites			
Neck	13 (10.2 %)	71 (14.3 %)	84 (13.5 %)
Armpits	0 (0 %)	2 (0.4 %)	2 (0.3 %)
Inguinal Area	0 (0 %)	2 (0.4 %)	2 (0.3 %)
Temple	0 (0 %)	10 (2.0 %)	10 (1.6 %)
**Water Insecurity**			
Use of non-improved water as main water source for:			
Injecting drugs	9 (6.8 %)	40 (8.0 %)	49 (7.7 %)
Cleaning wounds	9 (6.8 %)	32 (6.5 %)	41 (6.5 %)
Handwashing	7 (5.3 %)	27 (5.3 %)	34 (5.3 %)
**Hygeine Insecurity**			
Basic hand hygiene insecurity	85 (63.9 %)	299 (58.9 %)	384 (59.9 %)
Bathing insecurity (<5 showers in the last week)	80 (60.2 %)	264 (52.0 %)	346 (53.7 %)
Number of showers in the last week			
Mean (SD)	4.1 (2.6)	4.5 (2.6)	4.4 (2.6)
Median [IQR]	3.0 [2.0, 7.0]	4.0 [2.0, 7.0]	4.0 [2.0, 7.0]
**Sanitation Insecurity**			
Basic sanitation insecurity (non-improved toilet facilities)			
No facility/open defecation	28 (21.1 %)	67 (13.1 %)	95 (14.8 %)
Unimproved toilet	4 (3.0 %)	12 (2.4 %)	16 (2.5 %)
Open defecated at least once in the last week	65 (48.9 %)	179 (35.1 %)	244 (37.9 %)
Times defecating in the open in the last week			
Mean (SD)	5.63 (5.34)	5.22 (4.28)	5.3 (4.6)
Median [IQR]	4.0 [2.0, 7.0]	4.0 [2.0, 7.0]	4.0 [2.0, 7.0]

Table 1 summarizes demographic and WASH insecurity variables among study participants at baseline, stratified by if an abscess was present during the baseline visit.

Note that *n* = 3 individuals were missing abscess information at baseline, *n* = 7 values were missing for years of education and *n* = 21 values were missing for the most common site of injection.

## References

[R1] Armenta RF, Roth AM, Wagner KD, Strathdee SA, Brodine SK, Cuevas-Mota J, … Garfein RS (2015). Prevalence and correlates of the use of prefilled syringes among persons who inject drugs in San Diego, CA. Journal of Urban Health, 92(6), 1081–1091. 26382653 10.1007/s11524-015-9988-6 PMC4675744

[R2] Avelar Portillo LJ, Calderón-Villarreal A, Abramovitz D, Harvey-Vera A, Cassels S, Vera CF, … Strathdee SA (2024). WaSH insecurity and anxiety among people who inject drugs in the Tijuana-San Diego border region. BMC Public Health, 24(1), 19. 38166866 10.1186/s12889-023-17341-9 PMC10763368

[R3] Avelar Portillo LJ, Kayser GL, Ko C, Vasquez A, Gonzalez J, Avelar DJ, … Chiang YY (2023). Water, sanitation, and hygiene (WaSH) insecurity in unhoused communities of Los Angeles, California. International Journal for Equity in Health, 22 (1), 108. 37264411 10.1186/s12939-023-01920-8 PMC10233557

[R4] Ballard AM, Cooper HLF, Young AM, & Caruso BA (2022). You feel how you look’: Exploring the impacts of unmet water, sanitation, and hygiene needs among rural people experiencing homelessness and their intersection with drug use. PLOS Water, 1(5), Article e0000019. 38742171 10.1371/journal.pwat.0000019 PMC11090493

[R5] Baltes A, Akhtar W, Birstler J, Olson-Streed H, Eagen K, Seal D, … Brown R (2020). Predictors of skin and soft tissue infections among sample of rural residents who inject drugs. Harm Reduction Journal, 17(1), 96. 33267848 10.1186/s12954-020-00447-3 PMC7709308

[R6] Bazzi AR, Harvey-Vera A, Buesig-Stamos T, Abramovitz D, Vera CF, Artamonova I, … Strathdee SA (2022). Study protocol for a pilot randomized controlled trial to increase COVID-19 testing and vaccination among people who inject drugs in San Diego County. Addiction Science & Clinical Practice, 17(1), 48. 36064745 10.1186/s13722-022-00328-z PMC9444113

[R7] Calderón-Villarreal A, Avelar Portillo LJ, Abramovitz D, Goldenberg S, Flanigan S, Quintana PJE, Harvey-Vera A, Vera CF, Rangel G, Strathdee SA, & Kayser GL (2024b). A brief instrument measuring the water, sanitation and hygiene domain of menstrual health among women who inject drugs. PloS One, 19(5), Article E0303378. 38728343 10.1371/journal.pone.0303378 PMC11086918

[R8] Calderón-Villarreal A, Avelar Portillo LJ, Abramovitz D, Goldenberg S, Flanigan S, Quintana PJE, Harvey-Vera A, Vera CF, Rangel G, Strathdee SA, & Kayser GL (2024a). Water, sanitation, and hygiene access among people who inject drugs in Tijuana and San Diego in 2020–2021: A cross-sectional study. International Journal for Equity in Health, 23(1), 79. 38644494 10.1186/s12939-024-02163-x PMC11034064

[R9] Calderón-Villarreal A, Terry B, Friedman J, González-Olachea SA, Chavez A, Díaz López M, (2022). Deported, homeless, and into the canal: Environmental structural violence in the binational Tijuana River. Social Science & Medicine 1982, 305, Article 115044. 35633600 10.1016/j.socscimed.2022.115044 PMC9585906

[R10] Chambers HF (2021). Skin and soft tissue infections in persons who inject drugs. Infectious Disease Clinics of North America, 35(1), 169–181. 33303334 10.1016/j.idc.2020.10.006

[R11] Colledge S, Larney S, Bruno R, Gibbs D, Degenhardt L, Yuen WS, … Peacock A (2020). Profile and correlates of injecting-related injuries and diseases among people who inject drugs in Australia. Drug and Alcohol Dependence, 216, 108267. 32916518 10.1016/j.drugalcdep.2020.108267

[R12] Cross L (2013). The classification and management of skin and soft tissue infections. International Emergency Nursing, 21(2), 84–88. 23615514 10.1016/j.ienj.2012.03.008

[R13] Dahlman D, Berge J, Björkman P, Nilsson AC, & Håkansson A (2018). Both localized and systemic bacterial infections are predicted by injection drug use: A prospective follow-up study in Swedish criminal justice clients. PloS One, 13(5), Article e0196944. 29851980 10.1371/journal.pone.0196944 PMC5979029

[R14] Dahlman D, Håkansson A, Kral AH, Wenger L, Ball EL, & Novak SP (2017). Behavioral characteristics and injection practices associated with skin and soft tissue infections among people who inject drugs: A community-based observational study. Substance Abuse : Research and Treatment, 38(1), 105–112. 10.1080/08897077.2016.1263592 27897966

[R15] DeMyers C, Warpinski C, & Wutich A (2017). Urban water insecurity: A case study of homelessness in Phoenix, Arizona. Environmental Justice, 10(3), 72–80.

[R16] Doran J, Harris M, Hope VD, Wright T, Edmundson C, Sinka K, & Heinsbroek E (2020). Factors associated with skin and soft tissue infections among people who inject drugs in the United Kingdom: A comparative examination of data from two surveys. Drug and Alcohol Dependence, 213, 108080. 32526658 10.1016/j.drugalcdep.2020.108080

[R17] Dwyer R, Topp L, Maher L, Power R, Hellard M, Walsh N, … Aitken C (2009). Prevalences and correlates of non-viral injecting-related injuries and diseases in a convenience sample of Australian injecting drug users. Drug and Alcohol Dependence, 100(1), 9–16. 19013725 10.1016/j.drugalcdep.2008.08.016

[R18] Fink DS, Lindsay SP, Slymen DJ, Kral AH, & Bluthenthal RN (2013). Abscess and self-treatment among injection drug users at four california syringe exchanges and their surrounding communities. Substance Use & Misuse, 48(7), 523–531. 23581506 10.3109/10826084.2013.787094 PMC4334130

[R19] Flanigan S, & Welsh M (2020). Unmet needs of individuals experiencing homelessness near San Diego waterways: The roles of displacement and overburdened service systems. Journal of Health and Human Services Administration, 43(2), 105–130.

[R20] Fraser H, Borquez A, Stone J, Abramovitz D, Brouwer KC, Goodman-Meza D, … Vickerman P (2021). Overlapping key populations and HIV transmission in Tijuana, Mexico: A modelling analysis of epidemic drivers. AIDS and Behavior, 25(11), 3814–3827. 34216285 10.1007/s10461-021-03361-2 PMC8560668

[R21] Friedman J, Montero F, Bourgois P, Wahbi R, Dye D, Goodman-Meza D, & Shover C (2022). Xylazine spreads across the US: A growing component of the increasingly synthetic and polysubstance overdose crisis. Drug and Alcohol Dependence, 233, 109380. 35247724 10.1016/j.drugalcdep.2022.109380 PMC9128597

[R22] Friedman J, & Shover CL (2023). Charting the fourth wave: Geographic, temporal, race/ethnicity and demographic trends in polysubstance fentanyl overdose deaths in the United States, 2010–2021. Addiction (Abingdon, England), 118(12), 2477–2485. 37705148 10.1111/add.16318

[R23] Gilbert AR, Hellman JL, Wilkes MS, Rees VW, & Summers PJ (2019). Self-care habits among people who inject drugs with skin and soft tissue infections: A qualitative analysis. Harm Reduction Journal, 16(1), 69. 31831010 10.1186/s12954-019-0345-z PMC6909440

[R24] Harris M, Brathwaite R, McGowan CR, Ciccarone D, Gilchrist G, McCusker M, … Hope V (2018). Care and Prevent’: Rationale for investigating skin and soft tissue infections and AA amyloidosis among people who inject drugs in London. Harm Reduction Journal, 15(1), 23. 29739408 10.1186/s12954-018-0233-y PMC5941602

[R25] Harris M, Scott J, Hope V, Wright T, McGowan C, & Ciccarone D (2020). Navigating environmental constraints to injection preparation: The use of saliva and other alternatives to sterile water among unstably housed PWID in London. Harm Reduction Journal, 17(1), 24. 32276626 10.1186/s12954-020-00369-0 PMC7145770

[R26] JMP, UNICEF, WHO. Progress on household drinking water, sanitation and hygiene 2000–2022: Special focus on gender [Internet]. 2023 [cited 2023 Dec 19]. Available from: https://washdata.org/reports/jmp-2023-wash-households.

[R27] Karimi M, Ghaheri H, Assari S, Moghani Lankarani R, Moghani Lankarani M, Rafiey H, Narenjiha H, Ahmedi K, Tavakoli M, & Jafari F (2014). Drug injection to sites other than arm: A study of Iranian heroin injectors. Frontiers in Psychiatry, 5(23). [Internet]Available from https://www.frontiersin.org/journals/psychiatry/articles/10.3389/fpsyt.2014.00023/full. 10.3389/fpsyt.2014.00023 PMC3985009 24778621

[R28] Kolla BP, Oesterle T, Gold M, Southwick F, & Rummans T (2020). Infectious diseases occurring in the context of substance use disorders: A concise review. Journal of the Neurological Sciences. [Internet][cited 2023 Aug 23];411. Available from https://www.jns-journal.com/article/S0022-510X(20)30055-1/fulltext. 10.1016/j.jns.2020.116719 32070807

[R29] Larney S, Peacock A, Mathers BM, Hickman M, & Degenhardt L (2017). A systematic review of injecting-related injury and disease among people who inject drugs. Drug and Alcohol Dependence, 171, 39–49. 28013096 10.1016/j.drugalcdep.2016.11.029

[R30] LavaMae. LavaMaex. 2022 [cited 2022 Sep 6]. Our Impact. Available from: https://lavamaex.org/impact.

[R31] Leibler JH, Liebschutz JM, Keosaian J, Stewart C, Monteiro J, Woodruff A, & Stein MD (2019). Homelessness, personal hygiene, and MRSA nasal colonization among persons who inject drugs. Journal of Urban Health: Bulletin of the New York Academy of Medicine, 96(5), 734–740. 31493182 10.1007/s11524-019-00379-9 PMC6814663

[R32] Lewer D, Hope V, Harris M, Kelleher M, Jewell A, Pritchard S, Strang J, & Morley KI (2020). Incidence and treatment costs of severe bacterial infections among people who inject heroin: A cohort study in South London, England. Drug and Alcohol Dependence, 212, Article 108057. 32422537 10.1016/j.drugalcdep.2020.108057 PMC7301433

[R33] Lewis R, & Asmus L (2019). People who inject drugs: Environmental assessment in San Diego [Internet]. San Diego: San Diego State University. Family Health Centers of San Diego; 2019 [cited 2022 Aug 18]. Available from https://view.officeapps.live.com/op/view.aspx?src=https%3A%2F%2Fwww.sandiegocounty.gov%2Fcontent%2Fdam%2Fsdc%2Fhhsa%2Fprograms%2Fphs%2Fhiv-planning-group%2FPWID%252010-7-20.pptx&wdOrigin=BROWSELINK.

[R34] Lipsker D (2021). An abscess is not a descriptive term but an entity with a universally accepted definition—a clarification on semantics. JAMA Dermatology, 157(10), 1244–1245. 34431985 10.1001/jamadermatol.2021.3169

[R35] Lloyd-Smith E, Kerr T, Hogg RS, Li K, Montaner JS, & Wood E (2005). Prevalence and correlates of abscesses among a cohort of injection drug users. Harm Reduction Journal, 2(1), 24. 16281979 10.1186/1477-7517-2-24 PMC1308840

[R36] Lloyd-Smith E, Wood E, Zhang R, Tyndall MW, Sheps S, Montaner JS, & Kerr T (2010). Determinants of hospitalization for a cutaneous injection-related infection among injection drug users: A cohort study. BMC Public Health, 10(1), 327. 20534148 10.1186/1471-2458-10-327 PMC2890691

[R37] McNeil JC, & Fritz SA (2019). Prevention strategies for recurrent community-associated *Staphylococcus aureus* skin and soft tissue infections. Current Infectious Disease Reports, 21(4), 12. 30859379 10.1007/s11908-019-0670-0

[R38] Neves-Silva P, Martins GI, Heller L “A gente tem acesso de favores, né?”. A percepção de pessoas em situação de rua sobre os direitos humanos à água e ao esgotamento sanitário. Cad Saúde Pública [Internet]. 2018 Mar 26 [cited 2020 May 7];34(3). Available from: http://www.scielo.br/scielo.php?script=sci_arttext&pid=S0102-311X2018000305019&lng=pt&tlng=pt. 10.1590/0102-311X00024017 29590241

[R39] Noroozi M, Armoon B, Ghisvand H, Noroozi A, Karimy M, Bazrafshan MR, … Dieji B (2019). Prevalence and risk factors for injection site skin infections among people who inject drugs (PWID) in Tehran. Journal of Cosmetic Dermatology, 18(1), 258–262. 29781249 10.1111/jocd.12675

[R40] Orange County. HMIS Related HUD Definitions [Internet]. 2014 [cited 2022 Jan 20]. Available from: http://www.ochmis.org/documents/file_HMIS_Related_HUD_Definitions.pdf.

[R41] O’Sullivan E, Nelson G, Aubry T, Laval C, Shinn M, Tsemberis S How social science can influence homelessness policy: Experiences from Europe, Canada, and the United States – Part II: Politics and policy change. 2021;15(2):29.

[R42] Otiashvili D, Latypov A, Kirtadze I, Ibragimov U, & Zule W (2016). Drug preparation, injection, and sharing practices in Tajikistan: A qualitative study in Kulob and Khorog. Substance Abuse Treatment, Prevention, and Policy, 11(1), 21. 27251514 10.1186/s13011-016-0065-2 PMC4890278

[R43] Ozga JE, Syvertsen JL, Zweifler JA, & Pollini RA (2022). A community-based study of abscess self-treatment and barriers to medical care among people who inject drugs in the United States. Health Social Care in the Community, 30(5), 1798–1808. 34469034 10.1111/hsc.13559 PMC8885857

[R44] Phillips-Howard PA, Osire E, Akinyi C, Zulaika G, Otieno FO, Mehta SD Water, sanitation and hygiene at sex work venues to support menstrual needs. Frontiers in Public Health [Internet]. 2024 [cited 2024 Mar 5];12. Available from: https://www.frontiersin.org/journals/public-health/articles/10.3389/fpubh.2024.1305601. 10.3389/fpubh.2024.1305601 PMC10936742 38481834

[R45] Pieper B (2019). Nonviral injection-related injuries in persons who inject drugs: Skin and soft tissue infection, vascular damage, and wounds. Advances in Skin & Wound Care, 32(7), 301. 31232837 10.1097/01.ASW.0000559612.06067.55

[R46] Pollini RA, Gallardo M, Hasan S, Minuto J, Lozada R, Vera A, … Strathdee SA (2010). High prevalence of abscesses and self-treatment among injection drug users in Tijuana, Mexico. The International Journal of Infectious Diseases, 14, e117–e122. 20381396 10.1016/j.ijid.2010.02.2238 PMC2917477

[R47] Robertson R, Broers B, & Harris M (2021). Injecting drug use, the skin and vasculature. Addiction (Abingdon, England), 116(7), 1914–1924. 33051902 10.1111/add.15283

[R48] Rosenberg CE (1966). Cholera in nineteenth-century Europe: A tool for social and economic analysis. Comparative Studies in Society and History, 8(4), 452–463.

[R49] Sanchez DP, Tookes H, Pastar I, & Lev-Tov H (2021). Wounds and skin and soft tissue infections in people who inject drugs and the utility of syringe service programs in their management. Advances in Wound Care : the Journal for Prevention and Healing, 10(10), 571–582. 10.1089/wound.2020.1243 PMC8312019 33913781

[R50] Strathdee SA, Abramovitz D, Harvey-Vera A, Vera CF, Rangel G, Artamonova I, … Petterson TL (2021). Prevalence and correlates of SARS-CoV-2 seropositivity among people who inject drugs in the San Diego-Tijuana border region. PloS One, 16 (11), Article e0260286. 34807963 10.1371/journal.pone.0260286 PMC8608290

[R51] Strathdee SA, Magis-Rodriguez C, Mays VM, Jimenez R, & Patterson TL (2012). The emerging HIV epidemic on the Mexico-US border: An international case study characterizing the role of epidemiology in surveillance and response. Annals of Epidemiology, 22(6), 426–438. 22626001 10.1016/j.annepidem.2012.04.002 PMC3361703

[R52] Summers PJ, Struve IA, Wilkes MS, & Rees VW (2017). Injection-site vein loss and soft tissue abscesses associated with black tar heroin injection: A cross-sectional study of two distinct populations in USA. The International Journal of Drug Policy, 39, 21–27. 27768990 10.1016/j.drugpo.2016.08.006

[R53] Therneau T, Crowson C, Atkinson E Using time dependent covariates and time dependent coefficients in the cox model [Internet]. Mayo Clinic; 2023. Available from: https://cran.r-project.org/web/packages/survival/vignettes/timedep.pdf.

[R54] Twisk JWR (2013). Applied longitudinal data analysis for epidemiology. Cambridge University Press.

[R55] Uddin SMN, Walters V, Gaillard JC, Hridi SM, & McSherry A (2016). Water, sanitation and hygiene for homeless people. Journal of Water and Health, 14(1), 47–51. 26837829 10.2166/wh.2015.248

[R56] UN General Assembly. The human right to water and sanitation: Resolution /adopted by the General Assembly [Internet]. UN; 2010 Aug [cited 2022 Aug 8]. Available from: https://digitallibrary.un.org/record/687002.

[R57] US Department of Housing and Urban Development. (2021). In.

[R58] Verbyla ME, Calderon JS, Flanigan S, Garcia M, Gersberg R, Kinoshita AM, … Welsh M (2021). An assessment of ambient water quality and challenges with access to water and sanitation services for individuals experiencing homelessness in riverine encampments. Environmental Engineering Science, 38(5), 389–401. 34079210 10.1089/ees.2020.0319 PMC8165467

[R59] West BS, Abramovitz DA, Gonzalez-Zuniga P, Rangel G, Werb D, Cepeda J, … Strathdee SA (2020). Drugs, discipline and death: Causes and predictors of mortality among people who inject drugs in Tijuana, 2011–2018. The International Journal of Drug Policy, 75, 102601. 31775080 10.1016/j.drugpo.2019.11.009 PMC6957706

[R60] WHO. Progress on sanitation and drinking water: 2015 update and MDG assessment [Internet]. 2015 [cited 2022 Aug 8]. Available from: https://www.who.int/publications-detail-redirect/9789241509145.

[R61] WHO, UNICEF. Joint Monitoring Program (JMP) [Internet]. 2020 [cited 2020 Dec 14]. Available from: https://washdata.org/.

[R62] Wright T, Hope V, Ciccarone D, Lewer D, Scott J, & Harris M (2020). Prevalence and severity of abscesses and cellulitis, and their associations with other health outcomes, in a community-based study of people who inject drugs in London, UK. PloS One, 15(7), Article e0235350. 32663203 10.1371/journal.pone.0235350 PMC7360031

[R63] Wurcel AG, Burke D, Skeer M, Landy D, Heimer R, Wong JB, … Stopka TH (2018). Sex work, injection drug use, and abscesses: Associations in women, but not men. Drug and Alcohol Dependence, 185, 293–297. 29482054 10.1016/j.drugalcdep.2017.12.028 PMC5991097

[R64] Zelayandia E, & Leon M (2020). Homeless mobile hygiene in the community [Internet]. [Northridge]. Northridge: California State University. [cited 2022 Apr 12]. Available from https://scholarworks.csun.edu/bitstream/handle/10211.3/216082/Zelayandia-Elizabeth-thesis-2020.pdf?sequence=1.

