# Supplementary material for: Water, sanitation and hygiene insecurity predict abscess incidence among people who inject drugs in a binational US–Mexico metropolitan area: A longitudinal cohort study
Source: Int J Drug Policy. Author manuscript; Available in PMC 2026 Mar 31. (PMC13036794; doi:10.1016/j.drugpo.2024.104485)
Supplement: Supplementary material [file NIHMS2147403-supplement-Supplementary_material.docx]

**Supplemental Table 1.** Demographic comparison of *La Frontera* participants included in analysis (defined as participants participated in two or more follow-up surveys) vs. those excluded from analysis, 2020-2023

| **Variable** | **Included** | **Excluded** | **Overall** |
| --- | --- | --- | --- |
|  | **(N=647)** | **(N=73)** | **(N=720)** |
| **Age** |  |  |  |
| Mean (SD) | 43.4 (10.8) | 39.8 (11.2) | 43.0 (10.8) |
| Median [Min, Max] | 43.0 [20.0, 72.0] | 38.0 [20.0, 65.0] | 42.0 [20.0, 72.0] |
| 45+ years | 287 (44.4%) | 27 (37.0%) | 314 (43.6%) |
| <45 years | 360 (55.6%) | 46 (63.0%) | 406 (56.4%) |
| **City of Residence** |  |  |  |
| Tijuana | 196 (30.3%) | 6 (8.2%) | 202 (28.1%) |
| San Diego | 451 (69.7%) | 67 (91.8%) | 518 (71.9%) |
| **Years of Education** |  |  |  |
| Mean (SD) | 10.0 (3.17) | 11.5 (3.18) | 10.2 (3.20) |
| Median [Min, Max] | 11.0 [1.0, 20.0] | 12.0 [4.0, 30.0] | 11.0 [1.0, 30.0] |
| Missing | 7 (1.1%) | 0 (0%) | 7 (1.0%) |
| <10 years | 253 (39.1%) | 11 (15.1%) | 264 (36.7%) |
| 10+ years | 387 (59.8%) | 62 (84.9%) | 449 (62.4%) |
| Missing | 7 (1.1%) | 0 (0%) | 7 (1.0%) |
| **Gender** |  |  |  |
| Men | 463 (71.6%) | 58 (79.5%) | 521 (72.4%) |
| Women | 178 (27.5%) | 15 (20.5%) | 193 (26.8%) |
| Nonbinary | 5 (0.8%) | 0 (0%) | 5 (0.7%) |
| Trans Men | 1 (0.2%) | 0 (0%) | 1 (0.1%) |
| Trans Women | 0 (0%) | 0 (0%) | 0 (0%) |
| **Housing Status** |  |  |  |
| Permanent housing | 184 (28.4%) | 5 (6.8%) | 189 (26.3%) |
| Homelessness |  |  |  |
| Sheltered | 123 (19.0%) | 7 (9.6%) | 130 (18.1%) |
| Unsheltered | 340 (52.6%) | 61 (83.6%) | 401 (55.7%) |
| **Sex Work Status** |  |  |  |
| No sex work | 594 (91.8%) | 71 (97.3%) | 665 (92.4%) |
| Sex work | 53 (8.2%) | 1 (1.4%) | 54 (7.5%) |
| Missing | 0 (0%) | 1 (1.4%) | 1 (0.1%) |

**Supplemental Table 2.** Missing data for each WASH insecurity variables before and after imputation by visit and overall

|  |  |  |  |  |  |  |
| --- | --- | --- | --- | --- | --- | --- |
| **WASH Insecurity Variable** | **Visit 1 (N=647)** | **Visit 2 (N=601)** | **Visit 3 (N=573)** | **Visit 4 (N=488)** | **Visit 5 (N=440)** | **Overall* (N=2,749)** |
| Use of non-improved water sources for preparing drugs for injection |  |  |  |  |  |  |
| Non-imputed | 16 (2.5%) | 24 (4.0%) | 489 (85.3%) | 164 (33.6%) | 17 (3.9%) | 710 (25.8%) |
| Imputed | 10 (1.5%) | 18 (3.0%) | 7 (1.2%) | 12 (2.5%) | 3 (0.7%) | 50 (1.8%) |
| Use of non-improved water sources for cleaning wounds |  |  |  |  |  |  |
| Non-imputed | 22 (3.4%) | 517 (86.0%) | 485 (84.6%) | 153 (31.4%) | 9 (2.0%) | 1,186 (43.1%) |
| Imputed | 15 (2.3%) | 9 (1.5%) | 12 (2.1%) | 145 (29.7%) | 2 (0.5%) | 183 (6.7%) |
| Use of non-improved water sources for handwashing |  |  |  |  |  |  |
| Non-imputed | 8 (1.2%) | 3 (0.5%) | 484 (84.5% | 144 (29.5%) | 0 (0%) | 639 (23.2%) |
| Imputed | 1 (0.2%) | 0 (0%) | 1 (0.2%) | 1 (0.2%) | 0 (0%) | 3 (0.1%) |
| Basic hand hygiene insecurity |  |  |  |  |  |  |
| Non-imputed | 102 (15.8%) | 6 (1.0%) | 484 (84.5%) | 144 (29.5%) | 0 (0%) | 736 (26.8%) |
| Imputed | 3 (0.5%) | 2 (0.3%) | 1 (0.2%) | 1 (0.2%) | 0 (0%) | 7 (0.3%) |
| Bathing insecurity |  |  |  |  |  |  |
| Non-imputed | 10 (1.5%) | 5 (0.8%) | 485 (84.6%) | 144 (29.5%) | 0 (0%) | 738 (26.8%) |
| Imputed | 3 (0.5%) | 2 (0.3%) | 1 (0.2%) | 1 (0.2%) | 0 (0%) | 7 (0.3%) |
| Basic sanitation insecurity |  |  |  |  |  |  |
| Non-imputed | 103 ( 15.9%) | 2 (0.3%) | 484 (84.5%) | 144 (29.5%) | 0 (0%) | 733 (26.7%) |
| Imputed | 1 (0.2%) | 0 (0%) | 1 (0.2%) | 2 (0.4%) | 0 (0%) | 4 (0.1%) |
| Open defecation |  |  |  |  |  |  |
| Non-imputed | 102 (15.8%) | 4 (0.7%) | 484 (84.5%) | 144 (29.5%) | 3 (0.7%) | 737 (26.8%) |
| Imputed | 1 (0.2%) | 0 (0%) | 1 (0.2%) | 2 (0.4%) | 0 (0%) | 4 (0.1%) |

*The overall N refers to the number of survey observations (and not the number of participants).

Supplemental Table 2 described the missing data for each visit. The remaining missing values were due to the imputation process being limited to 12 months, so values were not extended past 12 months from their collection date.

**Supplemental Table 3.** Testing Proportional Hazards Assumption of Cox Model

| **Variable** | **chisq** | **df** | **p value** |
| --- | --- | --- | --- |
| Bathing insecurity | 0.125 | 1 | 0.724 |
| Basic hand hygiene insecurity | 2.787 | 1 | 0.095 |
| Days since last injection (one standard deviation increase) | 0.317 | 1 | 0.573 |
| Basic sanitation insecurity | 0.018 | 1 | 0.892 |
| Non-improved water for injecting drugs | 1.680 | 1 | 0.195 |
| Open defecation | 3.055 | 1 | 0.080 |
| Injects in safer body areas | 0.893 | 1 | 0.345 |

Supplemental table 3 shows the results of testing for the proportionality assumption of each variable included in the Cox model with time dependent covariates. Although the model includes time dependent covariates, it does still assume proportionality, i.e. that the effect of those covariates does not vary over time. Following the methodology proposed by Grambsch and Therneau (see below), we assessed proportionality, using the cox.zph() function in the *survival*  package in R. For each variable, the p value was above 0.05 indicating a lack of evidence of non-proportionality. Therefore, the use of time-varying coefficients (which would add significant complexity to the model) was deemed to be not necessary. See: *P. Grambsch and T. Therneau (1994), Proportional hazards tests and diagnostics based on weighted residuals.*Biometrika,*81, 515-26.*

**Supplemental Table 4.** Abscess incidence rates and unadjusted and adjusted hazard ratios by WASH variables and demographic covariates

| **Variable** | **Level** | **Person Years** | **Abscesses Observed** | **Incidence Rate per 100 person-year (95%CI)** | **Unadjusted Hazard Ratio (95%CI)** | **Unadjusted p value** | **Adjusted Hazard Ratio (95%CI)** | **Adjusted p value** |
| --- | --- | --- | --- | --- | --- | --- | --- | --- |
| All |  | 784 | 191 | 24.4 (21.1 - 27.6) |  |  |  |  |
| **WASH Variables** | | | | | | | | |
| Non-Improved Water for  Injecting Drugs | Yes | 61 | 30 | 49.6 (34.2 - 65.0) | 1.85 (1.28 - 2.68) | 0.001* | 1.49 (1.01 - 2.21) | 0.045* |
|  | No | 714 | 161 | 22.6 (19.3 - 25.8) |  |  |  |  |
| Non-Improved Water for  Cleaning Wounds | Yes | 65 | 23 | 35.7 (22.4 - 48.9) | 1.50 (0.98 - 2.29) | 0.062 |  |  |
|  | No | 665 | 159 | 23.9 (20.4 - 27.4) |  |  |  |  |
| Non-Improved Water for  Handwashing | Yes | 40 | 12 | 30.0 (14.4 - 45.6) | 1.33 (0.73 - 2.41) | 0.354 |  |  |
|  | No | 744 | 179 | 24.1 (20.8 - 27.4) |  |  |  |  |
| Basic Hygiene Insecurity (Handwashing without water/soap) | Yes | 518 | 129 | 24.9 (20.9 - 29.0) | 1.83 (1.33 - 2.51) | p<0.001* | 1.21 (0.86 - 1.70) | 0.284 |
|  | No | 265 | 62 | 23.4 (17.9 - 28.9) |  |  |  |  |
| Bathing Insecurity | Yes | 367 | 108 | 29.4 (24.3 - 34.6) | 2.16 (1.58 - 2.93) | p<0.001* | 1.59 (1.12 - 2.24) | 0.009* |
|  | No | 416 | 83 | 20.0 (15.9 - 24.1) |  |  |  |  |
| Basic Sanitation Insecurity (Non-improved toilet facilities) | Yes | 65 | 22 | 33.8 (21.0 - 46.7) | 1.64 (1.04 - 2.57) | 0.033* | 0.72 (0.42 - 1.21) | 0.210 |
|  | No | 718 | 169 | 23.5 (20.2 - 26.9) |  |  |  |  |
| Open Defecation | Yes | 244 | 104 | 42.6 (35.4 - 49.9) | 2.22 (1.67 - 2.97) | p<0.001* | 1.65 (1.16 - 2.35) | 0.005* |
|  | No | 539 | 87 | 16.1 (12.9 - 19.4) |  |  |  |  |
| **Potential Confounders** | | | | | | | | |
| Age 45+ | Yes | 392 | 96 | 24.5 (19.9 - 29.1) | 1.08 (0.81 - 1.44) | 0.603 |  |  |
|  | No | 393 | 95 | 24.2 (19.6 - 28.8) |  |  |  |  |
| <10 Years of Education | Yes | 354 | 95 | 26.9 (21.8 - 31.9) | 1.32 (0.98 - 1.78) | 0.066 |  |  |
|  | No | 420 | 94 | 22.4 (18.1 - 26.6) |  |  |  |  |
| Residence in Tijuana | Yes | 543 | 128 | 23.6 (19.7 - 27.4) | 1.24 (0.89 - 1.72) | 0.208 |  |  |
|  | No | 241 | 63 | 26.1 (20.1 - 32.2) |  |  |  |  |
| Female Gender | Yes | 221 | 46 | 20.8 (15.1 - 26.5) | 0.77 (0.54 - 1.09) | 0.145 |  |  |
|  | No | 559 | 145 | 26.0 (22.0 - 29.9) |  |  |  |  |
| Homelessness | Yes | 612 | 153 | 25.0 (21.3 - 28.7) | 1.21 (0.84 - 1.75) | 0.298 |  |  |
|  | No | 173 | 38 | 22.0 (15.4 - 28.6) |  |  |  |  |
| Reported Sex Work | Yes | 36 | 8 | 22.2 (7.7 - 36.7) | 0.76 (0.37 - 1.54) | 0.439 |  |  |
|  | No | 748 | 183 | 24.5 (21.1 - 27.8) |  |  |  |  |
| Injects in More Dangerous Body Areas | Yes | 173 | 55 | 31.8 (24.1 - 39.5) | 1.76 (1.29 - 2.40) | p<0.001* | 1.38 (1.01 - 1.88) | 0.041* |
|  | No | 607 | 136 | 22.4 (18.9 - 26.0) |  |  |  |  |
| Days since last time injection  (One standard deviation increase) |  |  |  |  | 0.72 (0.55 - 0.94) | 0.017* | 0.83 (0.65 - 1.07) | 0.146 |

* Significant value (p<0.05)

Supplemental Table 4 shows precise numerical values for the results shown visually in Figures 1 and 2, including incidence rates, and adjusted and unadjusted hazard ratios, for all predictors assessed.


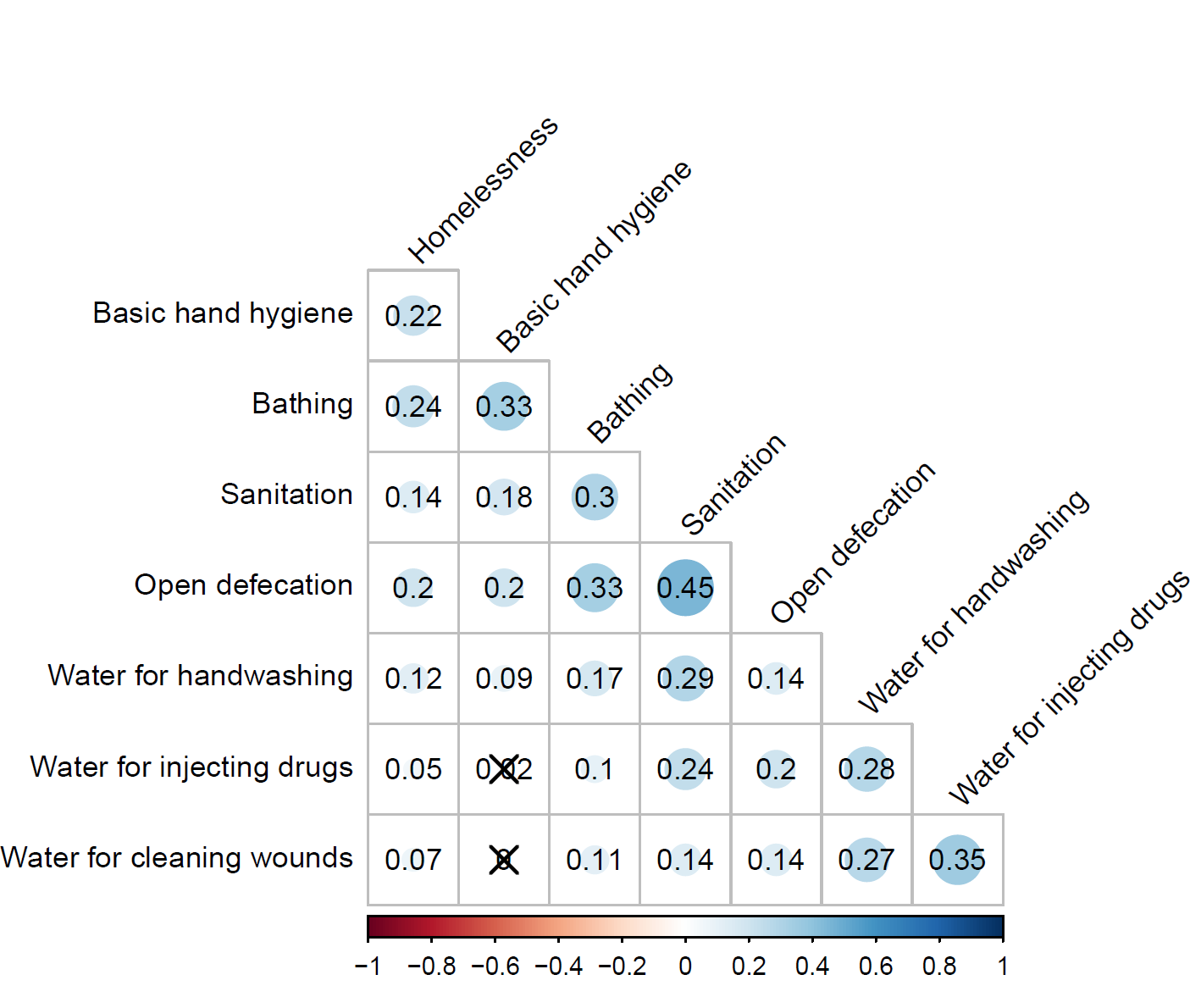
**Supplemental Figure 1.** WASH insecurity variables correlations matrix

In Supplemental Figure 1 we assess the model for multi-collinearity between WASH predictors and homelessness using a correlation matrix. The size of each bubble indicates the strength of the positive correlation. An X marks the non-significant Spearman correlations (at the .05 level). Overall, low to moderate correlations were seen between each WASH variable, indicating that collinearity was not present. Each WASH variable was therefore considered for inclusion in the final adjusted model. We explored correlations by visit with similar results.
